# Identification of the sex-determining locus in grass puffer (*Takifugu niphobles*) provides evidence for sex-chromosome turnover in a subset of *Takifugu* species

**DOI:** 10.1371/journal.pone.0190635

**Published:** 2018-01-02

**Authors:** Risa Ieda, Sho Hosoya, Shota Tajima, Kazufumi Atsumi, Takashi Kamiya, Aoi Nozawa, Yuma Aoki, Satoshi Tasumi, Takashi Koyama, Osamu Nakamura, Yuzuru Suzuki, Kiyoshi Kikuchi

**Affiliations:** 1 Fisheries Laboratory, University of Tokyo, Hamamatsu, Shizuoka, Japan; 2 School of Marine Biosciences, Kitasato University, Sagamihara, Kanagawa, Japan; Leibniz-Institute of Freshwater Ecology and Inland Fisheries, GERMANY

## Abstract

There is increasing evidence for frequent turnover in sex chromosomes in vertebrates. Yet experimental systems suitable for tracing the detailed process of turnover are rare. In theory, homologous turnover is possible if the new sex-determining locus is established on the existing sex-chromosome. However, there is no empirical evidence for such an event. The genus *Takifugu* includes fugu (*Takifugu rubripes*) and its two closely-related species whose sex is most likely determined by a SNP at the *Amhr2* locus. In these species, males are heterozygous, with G and C alleles at the SNP site, while females are homozygous for the C allele. To determine if a shift in the sex-determining locus occurred in another member of this genus, we used genetic mapping to characterize the sex-chromosome systems of *Takifugu niphobles*. We found that the G allele of *Amhr2* is absent in *T*. *niphobles*. Nevertheless, our initial mapping suggests a linkage between the phenotypic sex and the chromosome 19, which harbors the *Amhr2* locus. Subsequent high-resolution analysis using a sex-reversed fish demonstrated that the sex-determining locus maps to the proximal end of chromosome 19, far from the *Amhr2* locus. Thus, it is likely that homologous turnover involving these species has occurred. The data also showed that there is a male-specific reduction of recombination around the sex-determining locus. Nevertheless, no evidence for sex-chromosome differentiation was detected: the reduced recombination depended on phenotypic sex rather than genotypic sex; no X- or Y-specific maker was obtained; the YY individual was viable. Furthermore, fine-scale mapping narrowed down the new sex-determining locus to the interval corresponding to approximately 300-kb of sequence in the fugu genome. Thus, *T*. *niphobles* is determined to have a young and small sex-determining region that is suitable for studying an early phase of sex-chromosome evolution and the mechanisms underlying turnover of sex chromosome.

## Introduction

The sex chromosome and the sex-determining gene within have been maintained in therian mammals and birds for more than one hundred million years [1,2]. However, such stability and conservation of sex-determining system across a wide range of taxa is not universal in vertebrates. For example, sex chromosomes are not necessarily orthologous across many taxa in fish, amphibians and non-avian reptiles, and there even appears to be frequent non-homologous turnover in these chromosomes [3–8].

It has been shown that non-homologous turnover of sex chromosomes occurs through at least three mechanisms in vertebrates: (1) by the acquisition of a new sex-determining gene on an autosome [9–11], (2) by the transposition of an existing sex-determining gene to an autosome [12], and (3) by fusion between an autosome and an existing sex-chromosome [13,14]. In theory, reuse of the sex chromosomes, termed homologous turnover, is possible if the new sex-determining locus is established on an existing sex-chromosome through mechanisms (1) or (2). There is no empirical evidence for it in vertebrates [15].

While there is a broad literature on the evolutionary causes of sex-chromosome turnover (reviewed in [16,17], e.g. [18–20]), experimental systems suitable for testing these hypotheses or tracing the detailed process of turnover events are rare. Teleost fishes are a useful group of animals to investigate the process of sex-chromosome turnover and evolution of new sex-determining genes because different sex-determination mechanisms exist in closely related species [21,22], and the master sex-determining genes (or strong candidate genes) have been identified in some of these groups [23–30].

**Fig 1:**
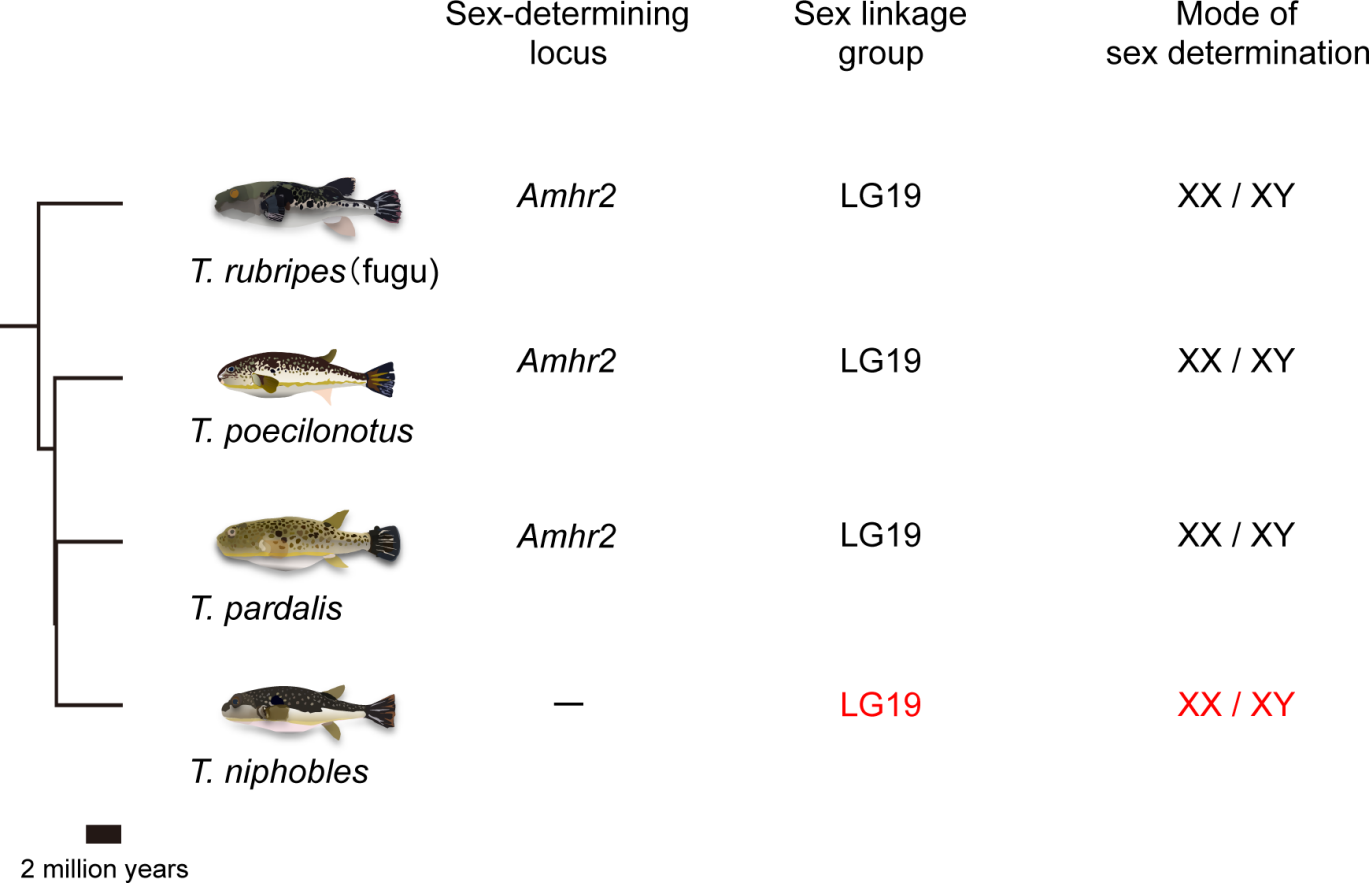
Phylogenetic relationship of four *Takifugu* species. The phylogeny is reproduced from [31] in which mitochondrial DNA data of 14 *Takifugu* and other related species have been analyzed. Also shown is a summary of previous results [26,33] and results from the current study (in red) on the sex-determining gene involved, sex linkage group, and the corresponding mode of heterogametic sex-determination for each species.

Among fishes, fugu (*Takifugu rubripes*) and its closely-related species hold promise for understanding the process of sex-chromosome turnover, for the following reasons. *Takifugu* underwent rapid speciation 2–5 million years ago, resulting in approximately 20 extant species [31,32]. Previous studies using a combination of linkage and association mapping have shown that a missense single-nucleotide polymorphism (SNP) in the exon9 at the *Amhr2* locus (SNP7271) is the sole polymorphism associated with phenotypic sex [26,33]. While males are heterozygous for G and C alleles at this SNP site, females are homozygous for the C allele in natural populations of fugu and its two closely-related species, *T*. *poecilonotus* and *T*. *pardalis*. Thus it is most likely that the SNP7271 at the *Amhr2* locus is associated with determining sex in these species with an XX/XY sex-determination system (Fig 1). Therefore, presence or absence of this ‘sex-determining SNP’ can be used as a marker to follow the transitions between different sex-determining systems efficiently. In addition, whole-genome sequencing and subsequent studies of fugu have suggested that many hundreds (>1500) of neutral genetic markers from the draft genome sequence are applicable to closely related species, and that overall synteny and gene order are well conserved among *Takifugu* species [34–37]. Thus, with the existence of many closely-related species, a known strong candidate gene for sex determination in a subset of species within the taxa, and the broad availability of genomic resources for the closely related species, the *Takifugu* genus offers advantages for genetic experiments of the evolution of the sex determination system.

In this study, we investigated another closely-related species of fugu, *T*. *niphobles* (grass puffer) to determine if it shares the mechanisms for determining its sex with fugu and the other two species (*T*. *poecilonotus* and *T*. *pardalis*). After surveying the polymorphism at the SNP7271 site of the *Amhr2* locus in a natural population of *T*. *niphobles*, we performed genetic mapping of the sex-determining locus, which included a high-resolution analysis using a sex-reversed fish and its progeny. Based on our findings, we conclude that *T*. *niphobles* has a sex-determining locus that is distinct from that in fugu and its two closely related species, despite having sex chromosomes that are homologous in these species.

## Materials and methods

### Ethics statement

Experiments were approved by the IACUC (Institutional Animal Care and Use Committee) of the Graduate School of Agricultural and Life Sciences, University of Tokyo (P-170529004). All live fish were sacrificed by submersion in ice water for at least 10 minutes followed by decapitation.

### Sampling

We obtained samples of *T*. *niphobles* (7 females and 8 males) from Washidu port in Lake Hamana (34°43' N, 137°33' E), Japan, and fugu (*T*. *rubripes*) (8 females and 7 males) from Enshunada (30°16' N–34°40' N and 138°13' E–136°54' E), off-shore areas around the mid-western part of Japan. SNP data from *T*. *poecilonotus* (6 females and 7 males) and *T*. *pardalis* (8 females and 8 males) has been reported previously [26]. A natural population of *T*. *niphobles* was also sampled from Lake Hamana by catching 100 females and 100 males for the purpose of surveying *Amhr2* gene polymorphisms to determine if the missense SNP in exon 9 of the locus is present or absent at the population-level. Hamana fishermen's union is responsible for fisheries management in Hamana lake and part of Enshnada. The fish samples were obtained through the Central and Washizu branches of Hamana fishermen's union. No specific permission was required for this research as the samples were obtained through the official fishermen's unions. This study did not involve endangered or protected species.

### Sexing

Phenotypic sex was determined by observation under a dissecting microscope. When it was difficult to do so, the gonads were fixed in 10% formaldehyde at room temperature and sectioned at a thickness of 10 μm following paraffin embedding. Sections were stained with hematoxylin and eosin.

### DNA extraction

A small piece of the caudal fin was clipped and preserved in 600 ml of TNES Urea buffer (10 mM Tris-HCl, pH7.5, 125 mM NaCl, 10 mM EDTA, 1% sodium dodecyl sulfate, 8M urea) at room temperature [38]. Following Proteinase K treatment (55°C for 3 hours or 37°C for 12 hours), genomic DNA was extracted using the Gentra Puregene Tissue Kit (QIAGEN) according to the manufacturer’s protocol.

### *Amhr2* gene sequence comparison

The DNA containing exon 9 of the *Amhr2* gene and the flanking region was amplified as reported previously [26] with the exception that the primers, SD3exon8-2F (5’- TGGCTCCCAGCTCAGATTC-3’) and SD3exon10-2R (5’-TGCGTCCTGTGCGATTT-3’) were used. The DNA sequence of the PCR product was directly determined by Sanger’s method from both strands using Applied Biosystems 3130 genetic analyzer (Thermo Fisher) and BigDye version 3.1 Cycle Sequencing Kit (Thermo Fisher). The DNA containing the full-length coding region of *T*. *niphobles Amhr2* gene was amplified by PCR using KOD FX Neo (Toyobo) reagents suitable for amplifying large genomic fragments. Approximately 13 ng of genomic DNA was used as a template in 10 μl PCR reaction with the primer pair 33-1464k340F (5’-CTTCTCCAGTCTTTACCAGGAGTTTTACTT-3’) and 33-1464k13469R (5’-GCTCACAGAACCCTTCTCCTTTGTCTT-3’) (500 nM each). The cycling conditions were as follows: 94°C for 5 minutes, followed by 36 cycles of 94°C for 10 sec and 70°C for 12 min. The DNA sequence of the PCR product was directly determined using primers listed in S1 Table.

### High resolution melting (HRM) analysis

HRM assay was performed following the method of [39]. In brief, PCR was done with 0.5 μl of primers (10 nM), 1 μl of genomic DNA (10–30 ng/μl) and 4 μl of high-sensitivity master mix containing LCGreen fluorescent dye (Idaho Technology) in 10 μl reactions. The primer pair used was SA-SD3exon9SNPF (5’-CTCCTGGAAGGCTCTGT-3’) and SA-SD3exon9SNPR (5’-GCGTGCATCAGATACCATT-3’). The PCR protocol involved 95°C for 2 min, followed by 30 cycles of 94°C for 30 sec and 65°C for 30 sec. Following PCR, the reaction was heated to 94°C for 30 sec, and cooled to 25°C. The reaction was then heated at 0.1°C/second and fluorescence data during DNA melting was collected as the temperature rose from 50°C to 92°C using a Lightscanner (Idaho Technology).

### Experimental crosses

We produced two full-sib families (Families 1 and 2) from two wild breeding pairs for chromosome-wide linkage analysis (Table 1), and four half-sib families (Families 4–7) from four wild males and one wild female for recombination breakpoint analysis (S2 Table). Crossing was done by means of artificial insemination, and the water temperature maintained at 20°C until hatching. After hatching, the water temperature was gradually increased to 22°C over 5 days and kept constant until sampling. All the progeny was fed rotifers, *Artemia* nauplii and/or commercial pellets *ad libitum* depending on the developmental stage, and kept under a natural photoperiod.

**Table 1:** Numbers of fish showing concordance and discordance between phenotypic and genotypic sex among those used for genetic mapping.

Family name	Phenotypic male		Phenotypic female	
	Expected male genotype*	Expected female genotype*	Expected male genotype*	Expected female genotype*
Family 1	23 (XY)	2 (XX)	0 (XY)	14 (XX)
Family 2	15 (XY)	0 (XX)	8 (XY)	16 (XX)
Family 3**	77 (XY), 33 (YY)	1 (XX)	4 (XY), 0 (YY)	25 (XX)

* The expected male and female genotypes were determined based on haplotypes of three markers (f2004, f2003 and f2006) inherited from fathers.

** A mother suspected to have XY genotype was used.

In order to obtain offspring from individuals suspected to have undergone sex-reversal (XY females) in Family 1 or Family 2, we selected live fish with XY genotype at the marker loci near the sex-determining locus and raised them until eggs were laid. While keeping the juvenile fish individually in a labeled net cage, we prepared genomic DNA from their caudal fin and genotyped them for the markers f2004, f2003 and f2006 (S3 Table). We identified 19 XY individuals from 42 live fish in Family 1 and 15 XY individuals from 31 live fish in Family 2 without phenotypic information. We then raised these XY fish together in one tank for three years and selected candidates for spontaneous sex-reversals (potentially XY females) based on the bulginess of the abdomen and the absence of spermiation during the adult breeding season of April to May. The candidate fish were injected with approximately 150 μg/kg of luteinizing hormone-releasing hormone (Sigma-Aldrich) to induce ovulation. The eggs were artificially inseminated with cryopreserved sperm from a wild male following the method of Hosoya et al. [40]. After hatching, the water temperature was gradually increased from 20°C to 22°C over 5 days and kept constant until sampling. The progeny was fed rotifer, *Artemia* nauplii and/or commercial pellets *ad libitum* depending on the developmental stage, and were kept under a natural photoperiod.

### Genotyping of microsatellite markers

Procedures for genotyping of microsatellite markers are described elsewhere [36]. Fragment analysis was conducted using either the 4300 DNA Analysis system (LI-COR) or ABI3130 genetic analyzer and GeneMapper software (Life Technologies Corporation). Primer sequences for microsatellite markers and their genomic position in the draft genome sequence of fugu are listed in S3 Table.

### Genetic map construction

For each cross, genetic maps were built based on segregation data from the mother and the father independently. Ancestry-unknown markers were analyzed within a phase-known model of inbred pedigrees and converted to ancestry-known markers following the method of [41]. The linkage between markers and marker order were examined in each meiotic segregation event using the R/qtl software [42]. Kosambi’s mapping function was used to calculate genetic distances between markers. A combined map containing the maternal and the paternal segregation events was also constructed.

### Quantitative trait locus (QTL) analysis

QTL analysis was done using R/qtl [42] with a mapping step size of 0.1 cM using the expectation–maximization (EM) algorithm under a binary model. Chromosome-wide thresholds for significance (*P* < 0.001) were determined using 10,000 permutations. The 95% Bayesian confidence interval (CI) of the QTL location was estimated by means of *bayesint* function, and the percent phenotypic variance explained (PVE) by the QTL calculated using the *fitqtl* function in R/qtl.

### Comparative genomics

Linkage groups of *T*. *niphobles* were numbered based on synteny with the fugu linkage group numbering that was established in [35]. We used the fugu genome assembly (FUGU5/fr3, [36]) to estimate the physical length of marker intervals in the linkage map of *T*. *niphobles*, because previous studies have indicated that overall gene orders, synteny and karyotypes are well conserved between fugu and *T*. *niphobles* [37,43].

## Results

### Comparison of genomic sequences of the *Amhr2* gene among *T*. *niphobles* and other three closely-related species

**Fig 2:**
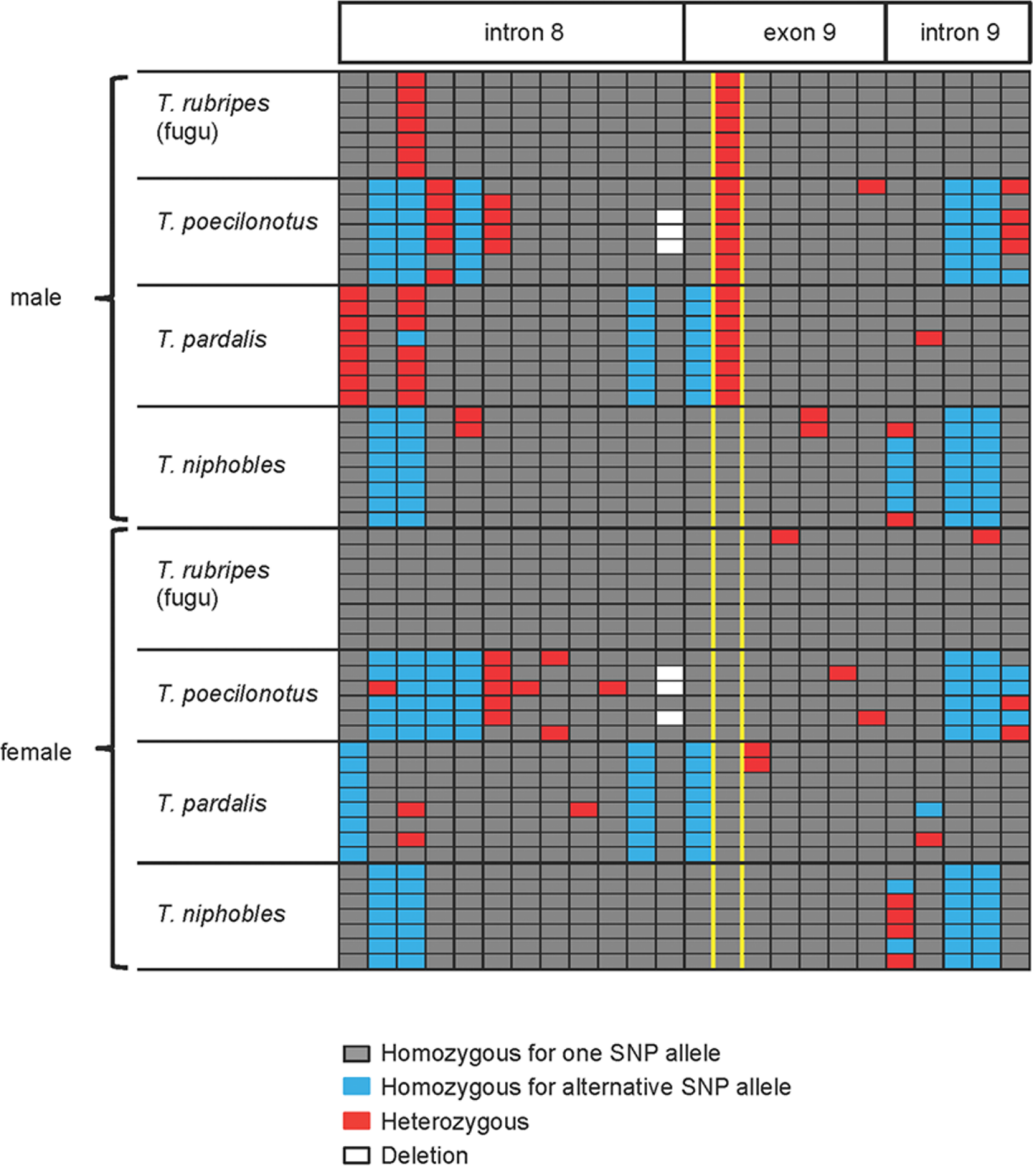
A comparison of SNPs around exon 9 of the *Amhr2* gene among four *Takifugu* species and between males and females. Shown are the 24 SNP sites detected in the partial sequence of the *Amhr2* gene (intron 8, exon 9, and intron 9). The association between the SNP7271 of the *Amhr2* gene (flanked by yellow lines) and phenotypic sex is conserved among fugu (*T*. *rubripes*), *T*. *poecilonotus* and *T*. *pardalis*, as reported previously [26,33], but not in *T*. *niphobles*. Gray cells indicate that an individual is homozygous for the reference allele, blue cells indicate that an individual is homozygous for the alternative allele, and red cells indicate that an individual is heterozygous. White cells refer to deletions.

Kamiya et al. [26] have suggested that a missense single-nucleotide polymorphism (SNP) at the *Amhr2* locus (SNP7271) is most likely to act as the sex-determining switch in fugu (*T*. *rubripes*) and its two closely-related species, *T*. *poecilonotus* and *T*. *pardalis*. In order to determine if another closely-related species of fugu, *T*. *niphobles*, shares the same mechanism for determining its sex, we compared the partial genomic sequences of the *Amhr2* gene (intron 8, exon 9 and intron 9) between female (*n* = 7) and male (*n* = 8) *T*. *niphobles*, and also compared them to the sequences from the corresponding sex in fugu, *T*. *poecilonotus* and *T*. *pardalis*. The comparison revealed that all the males from the three species other than *T*. *niphobles* were heterozygous (G/C) at the SNP7271 site (Fig 2, red cells flanked by yellow lines), while all females were homozygous for the C allele (Fig 2, grey cells flanked by yellow lines) as reported previously [26]. In contrast, all individuals of *T*. *niphobles* were found to be homozygous for the C allele at the SNP site, regardless of sex. These results suggest that the SNP7271 of the *Amhr2* gene does not determine sex in *T*. *niphobles*.

### A population-level survey for the sex-determining SNP in *Amhr2* in *T*. *niphobles*

**Fig 3:**
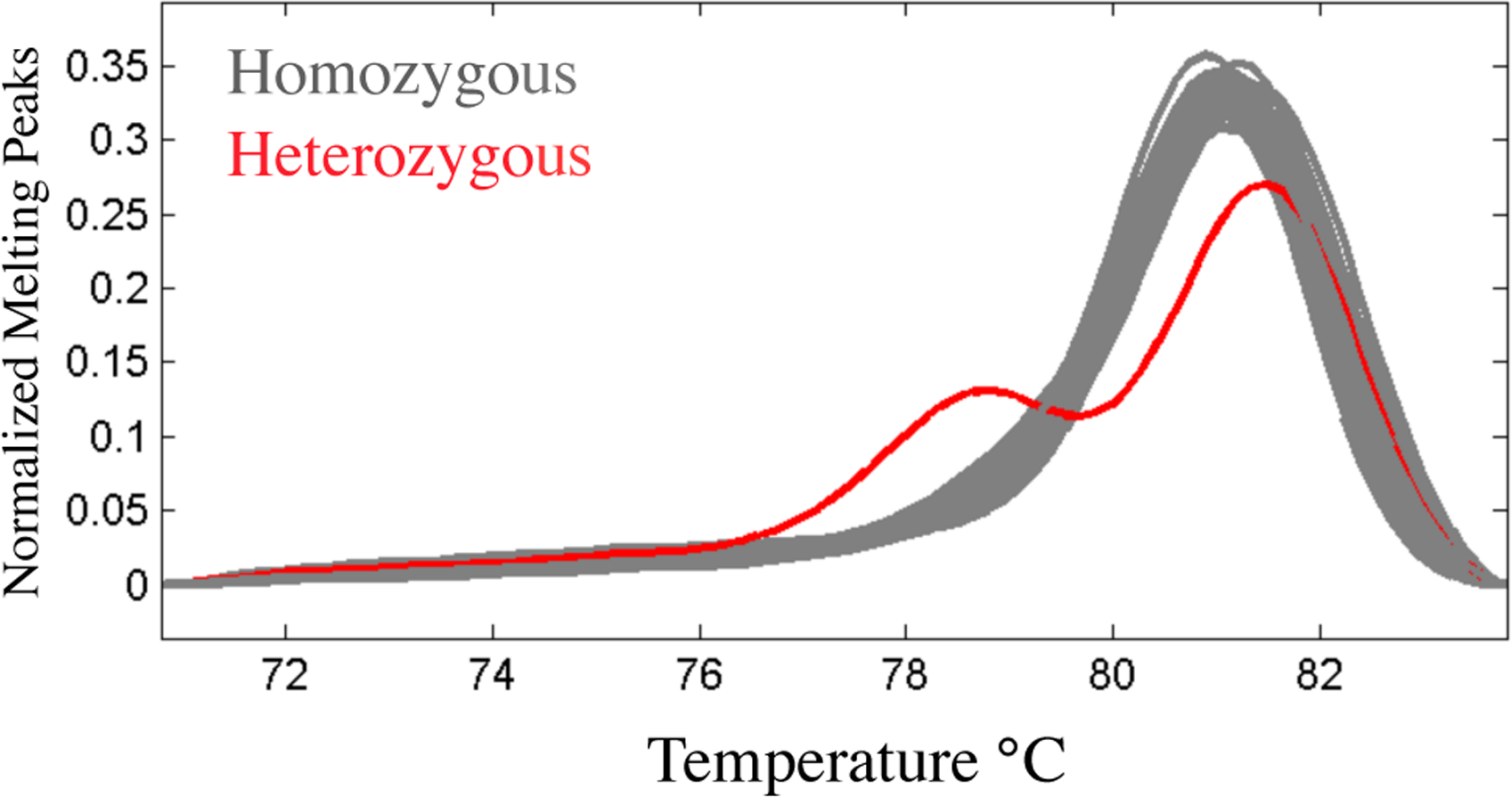
Derivative melting curves of PCR products by HRM analysis of SNP7271 at the *Amhr2* locus from fugu and *T*. *niphobles*. The red curve denotes a profile obtained from a male fugu heterozygous (XY) at the SNP site in exon 9 at the *Amhr2* locus (SNP7271). The 81 gray curves denote profiles obtained from 40 female and 40 male wild *T*. *niphobles*, and one female homozygous (XX) fugu. The same pattern (gray curves) was obtained from another 120 samples of *T*. *niphobles* (60 female and 60 male; data not shown), indicating that *T*. *niphobles* is homozygous at the SNP7271 position in the *Amhr2* gene regardless of the sex.

We conducted a population-level survey by determining the genotype at the SNP7271 site of the *Amhr2* gene of the 200 wild individuals of *T*. *niphobles* (*n* = 100 for each sex) from Lake Hamana using the high-resolution melting (HRM) assay developed by Matsunaga el al. [39]. This analysis revealed that all *T*. *niphobles* were homozygous at the SNP site regardless of their phenotypic sex (gray curves in Fig 3). The pattern in the melting curve seen in fugu XY males (red curve in Fig 3) was not shown by any *T*. *niphobles* male. Thus, unlike fugu, *T*. *poecilonotus* and *T*. *pardalis*, phenotypic sex in *T*. *niphobles* is likely to be determined by genomic region(s) other than the SNP7271 site at the *Amhr2* locus.

### Genetic mapping

**Fig 4:**
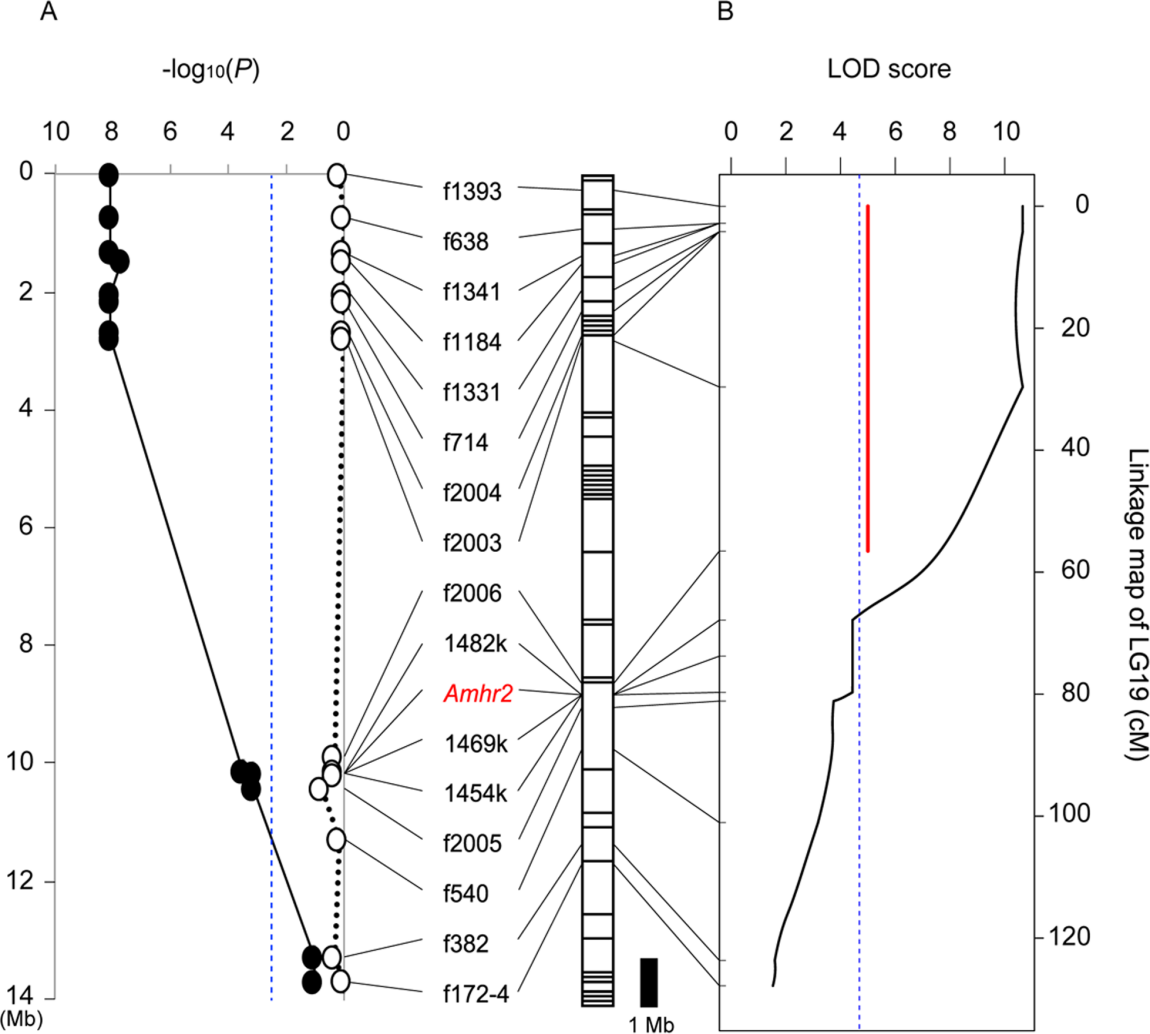
Association test for phenotypic sex and marker genotypes, and QTL (quantitative trait loci) analysis in *T*. *niphobles* Family 1. (A) Plot of–log_10_ (*P* value) versus chromosome position for the association test. The chromosomal position of the markers was first inferred from the draft genome sequence of fugu, and later confirmed partially by linkage analysis shown in Fig 4B and S1B Fig. Closed and open circles indicate data from paternally and maternally inherited markers, respectively. Bonferroni correction gave a significance threshold of–log_10_ (*P*) = 2.5 (blue vertical dotted line). The segmented bar next to the–log_10_ (*P*) plot illustrates the sequence map of fugu chromosome 19, in which each segment schematic represents a scaffold in the FUGU5/fr3 assembly [35,36]. (B) Chromosome-wide mapping of sex-determining QTL. Log of odds (LOD) scores are plotted in the linkage map of *T*. *niphobles* LG19. The blue dotted line indicates chromosome-wide significant (0.1%) levels of LOD scores, calculated from 10,000 permutations. The red line in the graph indicates the 95% Bayesian confidence interval (CI). Genetic markers are ordered and placed based on both the linkage analysis of *T*. *niphobles* (in the graph) and their comparative location in the fugu genome (on the segmented bar). There was no discrepancy in the order at this resolution of linkage analysis. The *Amhr2* locus (in red letters) did not co-segregate with 95% CI (red line).

In order to search for the sex-determining locus in *T*. *niphobles*, we analyzed the progeny produced from two independent genetic crosses (Families 1 and 2, Table 1) and tested for association between gonadal sex and marker genotypes. Since our preliminary experiments have suggested the linkage between sex determination and markers on LG (linkage group) 19 in *T*. *niphobles*, we focused on this linkage group, and found a strong association between sex and paternally inherited markers at or near the proximal end of LG19 in both families (Fig 4A, S1A Fig). The association decreased toward the distal end of LG19, and the markers near the *Amhr2* locus such as 1482k and 1469k showed intermediate association. There was no association between sex and maternally inherited markers in both families. These results suggested that a chromosome region at or near the proximal end of LG19 controls male or female sexual development in *T*. *niphobles* with an XX/XY system.

**Fig 5:**
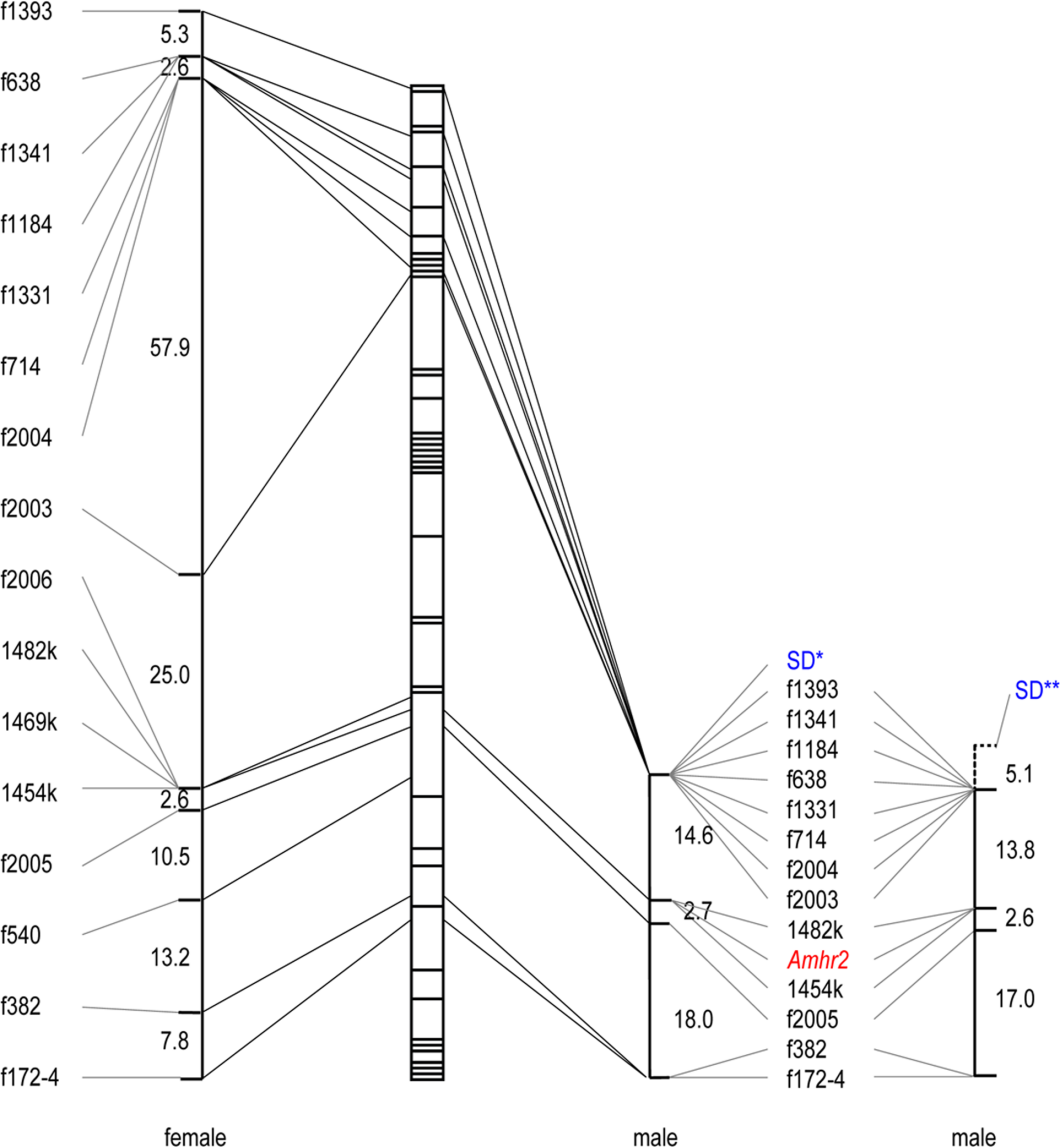
Female and male meiotic map of LG (linkage group) 19 in *T*. *niphobles* Family 1. Allelic bridges are indicated by a line connecting female (left) and male (right) linkage map. Genetic distances in centimorgans between adjacent markers are shown. Genetic markers are ordered and placed based on both the linkage analysis of *T*. *niphobles* and their comparative location in the fugu genome (on the segmented bar). There was no discrepancy in their order at this resolution of linkage analysis. Since it was not known if sex-reversed fish were present, two male maps were generated under these two conditions. SD* and SD** denote sex-determining locus.

However, there were mismatches between gonadal sex and genotypic sex even at the most strongly associated marker loci (Table 1). For example, two individuals among 25 phenotypic males in Family 1 exhibited the genotype expected of females, and eight individuals out of 24 females in Family 2 had the genotype expected of males. These mismatches could be due to incomplete penetrance of the master sex-determining gene. In other words, some offspring probably experienced sex-reversal with respect to the major sex-determining locus on LG19. Under this scenario, we excluded individuals suspected to have undergone sex-reversal from our data sets and performed linkage analysis based on recombination during meiosis in males. The analysis indicated that the sex-determining locus of *T*. *niphobles* maps to the proximal end of LG19 (shown as SD* in Fig 5 and S2 Fig). An alternative explanation for the mismatches is that our analysis failed to identify the markers that perfectly segregated with phenotypic sex on LG19. Under this assumption, we did not exclude the potentially sex-reversed fish and found that the major sex-determining locus was placed in the extended end of LG19 where no genetic makers have been developed (shown as SD** in Fig 5 and S2 Fig). In either scenario, the results so far implied that LG19 harbors the major sex-determining locus that may be different from the *Amhr2* locus in *T*. *niphobles*.

To conduct a more inclusive analysis, we treated sex in *T*. *niphobles* as a binary trait controlled by quantitative trait loci (QTL). This QTL analysis indicated the existence of a major QTL linked to LG19 for both families (Fig 4B and S1B Fig). The highest LOD (logarithm of odds) score for Families 1 and 2 was 10.7 and 9.32, respectively, and the location of the QTL for both families (Fig 4B, S1B Fig) largely overlapped that of the markers showing highest association with phenotypic sex in the association analysis (Fig 4A, S1A Fig). The phenotypic variance explained by the QTL for Families 1 and 2 was 73.5% and 75.0%, respectively. The 95% Bayesian confidence interval (CI) for the QTL location spanned a region of 56.5 cM and 53.6 cM in Families 1 and 2, respectively, corresponding to approximately 9.9 Mb in the draft sequence of fugu Chr19. While the highest LOD score of the QTL exceeded the chromosome-wide threshold of value of 4.69 (α = 0.001) in both families, the LOD score at the *Amhr2* locus did not. Moreover, the distal chip of the CI for the QTL location was separated from the *Amhr2* locus by 13.9 and 15.6 cM on the linkage map for Families 1 and 2, respectively. These results are consistent with the above-mentioned analyses (Figs 1–5) that suggest the dissociation between the major sex-determining locus and the *Amhr2* locus in *T*. *niphobles*.

### Recombination around the sex-determining gene in additional families

When the meiotic maps from the female and male parents were compared, large sex-specific differences were observed in both families (Fig 5 and S2 Fig). For example, in Family1, the distance between f1393 and f2003 in the female meiotic map was 65.8 cM, whereas it was zero cM in the male meiotic map. A similar discrepancy was observed in Family 2. These results suggested that there is a male-specific reduction in recombination around these sex-linked markers. To further confirm this tendency, we produced four families of *T*. *niphobles* composed of 502 siblings in total (Families 4–7, S2 Table) and searched for breakpoints between markers around the sex-determining locus in their male meiotic maps. Although we found 6 recombinants between the markers f383 and f1637 that span approximately 0.4 Mb of the genomic segment in fugu Chr19, we were not able to find any recombination between the markers f1637 and f714 that span approximately 3.7 Mb of the genomic segment in fugu Chr19 (S3 Fig). These results indicate that recombination around the sex-determining gene rarely occurs during meiosis in males of this species.

### Fine-scale mapping

The male-specific reduction in recombination around the sex-determining locus has hampered the precise localization of the causative/associated gene. To circumvent this problem, we took advantage of the possible occurrence of spontaneous sex-reversals, specifically, XY females. We anticipated that markers tightly linked to the male-determining gene would recombine during meiosis in phenotypic females that have the male-determining gene (XY females), as the frequency of recombination at the proximal end of LG19 was shown to be greater in females relative to males (Fig 5 and S2 Fig). A similar approach using artificially sex-reversed XY females was shown to be successful for fine mapping the male-determining gene in medaka [23].

**Fig 6:**
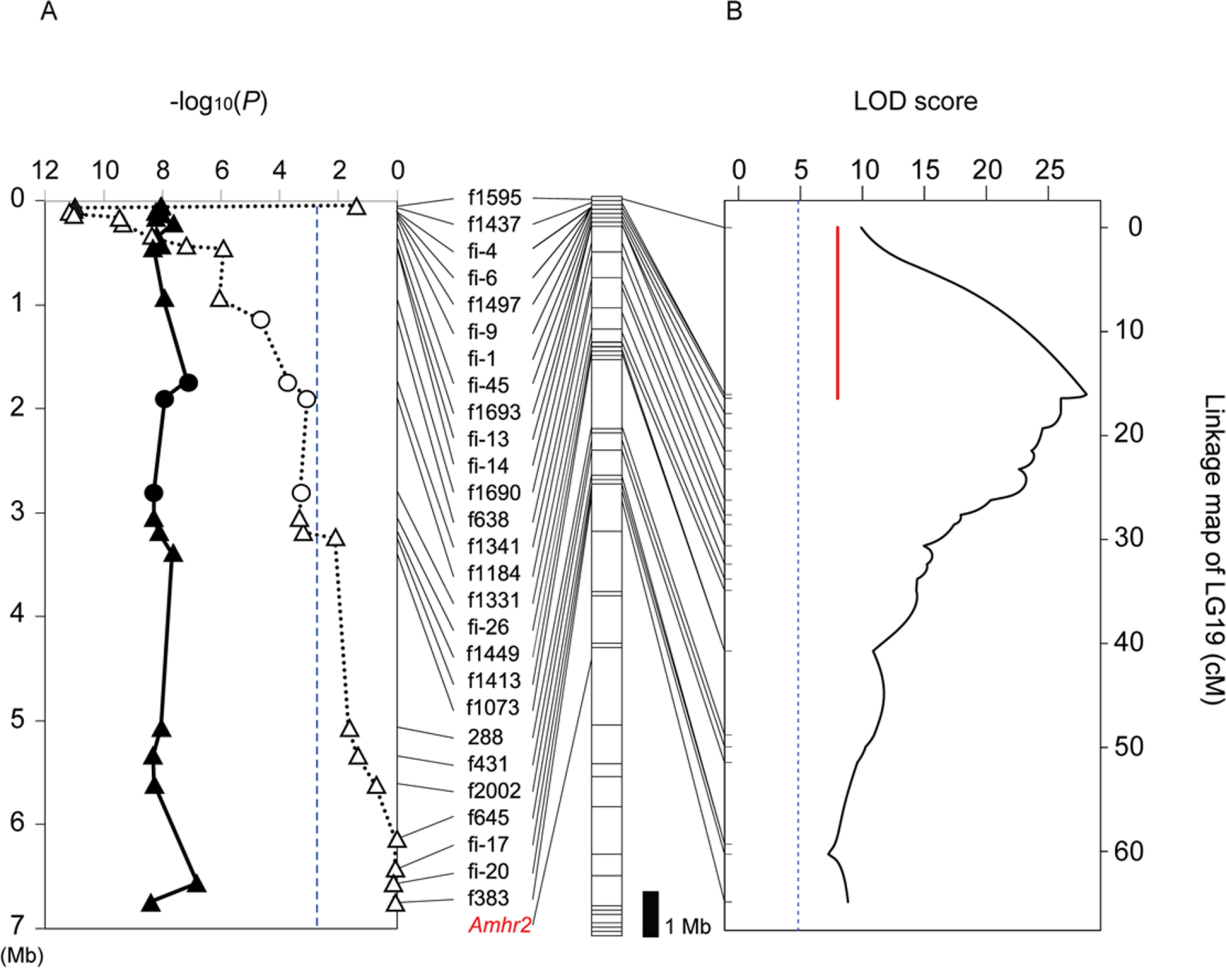
The sex-determining QTL in *T*. *niphobles* maps to a region distinct from the *Amhr2* locus in the high-resolution map. (A) Plot of–log_10_ (*P* value) versus chromosome position for association test using *T*. *niphobles* Family 3. Closed and open circles indicate data from paternally and maternally inherited markers, respectively, used in the initial analysis. Closed and open triangles indicate data from paternal and maternal inherited markers, respectively, added later. Bonferroni correction gave a significance threshold of–log_10_ (*P*) = 2.5 (blue vertical dotted line). The segmented bar next to the–log_10_ (*P*) plot illustrates the sequence map of fugu Chr19. (B) High-resolution mapping of sex-determining QTL. LOD scores are plotted in the linkage map of *T*. *niphobles* LG19. The blue dotted line indicates chromosome-wide significant (0.1%) levels of LOD scores, calculated from 10,000 permutations. The red line in the graph indicates the 95% Bayesian CI. Genetic markers are ordered based on both the linkage analysis of *T*. *niphobles* and their comparative location in the fugu genome. There was no discrepancy in their order at this resolution of linkage analysis.

We first selected XY fish from our experimental families based on the genotype at markers f2004, f2003 and f2006, and chose two individuals that appeared to be phenotypically female. After obtaining offspring by crossing one of the selected females with an XY male, we analyzed the phenotypic sex and marker genotypes of the progeny (Family 3 in Table 1). We found a strong association between phenotypic sex and maternally inherited markers around the sex-determining locus as well as with paternally inherited markers (Fig 6). This result clearly indicated that the mother carried the major male-determining gene on LG19, and had undergone spontaneous sex-reversal to become a fertile female.

Comparison of the meiotic map from the female and male parents revealed an increase in recombination events during meiosis in the mother relative to meiosis in the father (S4 Fig), as expected. In order to understand the detailed localization of male-determining locus in the *T*. *niphobles*, we first increased the marker density of the linkage map covering the proximal half of the sex chromosomes by referring to the draft genome assembly of fugu Chr19 (S3 Table, Fig 6). The resultant map of *T*. *niphobles* spanned 64.9 cM and consisted of 27 markers that anchored to the fugu scaffolds accounting for approximately 6.7 Mb in fugu Chr19. The linkage analysis using meiosis in the mother showed that the majority of these markers recombined each other, except for six markers, f1437, fi-4, fi-6, f1497, and fi-9 that span approximately 0.5 Mb of the genomic segment in fugu Chr19 (S4 Fig).

By using the high-resolution linkage map, we conducted a QTL analysis of the male-determining locus, and refined the localization of the major sex-determining region on LG19. The 95% Bayesian CI of the QTL location spanned a region of 16.4 cM (Fig 6), explaining 61.6% of the phenotypic variance. This result clearly demonstrated that the sex-determining locus in *T*. *niphobles* is different from the *Amhr2* locus that mapped to the middle of LG19. Thus the sex chromosomes of *T*. *niphobles* are homologous to those of fugu and the other two closely-related species, but *T*. *niphobles* does not share its sex-determining locus with them.

### Candidate genes

In order to identify candidates for the sex-determining gene in *T*. *niphobles*, we took advantage of the annotation of the fugu genome assembly. We first closely examined the fugu genome region corresponding to the *T*. *niphobles* sex-determining QTL by using the comparative location of genetic markers between species (Fig 6) and found that the genomic segment included at least five scaffolds with total length of 321 kb (Table 2). We then searched for genes known to be involved in sex determination and/or differentiation in other vertebrates [7,44]. Although there were 12 predicted protein coding-genes (Table 2) in the five scaffolds, we were not able to find any genes such as *Sox*, *Dmrt* or *TGF-β* genes that apparently play major roles in the sex determination/differentiation pathway. It should be noted that there were many unfilled gaps within these scaffolds, accounting for nearly 32% of the total length of the scaffolds (Table 2). Moreover, the length of inter-scaffold gaps was unknown. Therefore, many genes residing in the segment must be absent in the list shown in Table 2.

**Table 2:** Fugu scaffolds corresponding to the sex-determining QTL in *T*. *niphobles*.

Scaffold	Size (bp)	Undetermined sequences (bp)	Predicted protein cording-gene
586	73,234	13,395	ENSTRUG00000002032
617	64,632	24,672	ENSTRUG00000000092 (*Cyc1*)
			ENSTRUG00000000094 (*Ergic3*)
688	49,878	26,313	ENSTRUG00000002541 (*Appl1*)
			ENSTRUG00000003750 (*Il17rd*)
926	36,868	19,645	-
538	96,418	17,563	ENSTRUG00000012852
			ENSTRUG00000012861 (*C1qa*)
			ENSTRUG00000012869 (*Trim61*)
			ENSTRUG00000012884
			ENSTRUG00000012969 (*Ccdc72*)
			ENSTRUG00000012984 (*Ccdc51*)
			ENSTRUG00000013006 (*Osgin1*)

## Discussion

It has been shown that sex in fugu and its two closely-related species (*T*. *poecilonotus* and *T*. *pardalis*) is most likely determined by a combination of two allelic variants (the C and G alleles) at the *Amhr2* locus on their Chr19 [26]. Since *T*. *niphobles* is another closely-related species to fugu, but potentially has a different cue for the primary sex-determination, it may be illuminating to understand the turnover of sex-determining system among closely-related species of non-mammalian vertebrates.

By a series of genetic mapping, this study indicated that the sex-determining locus in *T*. *niphobles* is different from the *Amhr2* locus, despite the fact that these loci are located on the same chromosome (*T*. *niphobles* Chr19). A genetic survey of a wild population suggested that the *Amhr2* locus is fixed for the C allele. Analysis of recombination showed that the sex-determining locus is placed in a region where paternal recombination is strongly suppressed. Nevertheless, no evidence for the sex-chromosome degeneration was detected in this study. The fine mapping narrowed down the sex-determining locus to the interval corresponding to approximately 300-kb of sequence in the fugu genome, in which 12 genes were annotated.

### Homologous turnover of the sex chromosome

This study clearly shows that the sex-determining locus in *T*. *niphobles* is located near the proximal end of Chr19 (Fig 6). In conjunction with the previous study showing that the sex-determining locus in fugu mapped to the *Amhr2* locus in the middle of fugu Chr19 [26], the results suggested that at least two distinct sex-determining loci are established on homologous chromosomes (Chr19) in the *Takifugu* fishes and, thus, implied that homologous turnover of sex chromosome had occurred. Given the phylogenetic position of *T*. *niphobles* among 14 *Takifugu* species reported by Yamanoue et al. (2009) (see Fig 1), it appears that *T*. *niphobles* has obtained a derived sex-determining locus on Chr19, while having lost the ancestral male-determining allele (the G allele) at the *Amhr2* locus on Chr19. However, there are two major problems with this interpretation. The first is that the phylogenetic position shown by Yamanoue et al. (2009) was based on mitochondrial DNA data and some of the relationships among 14 *Takifugu* species were ambiguous [31]. Recent studies have shown the presence of historical hybridization between closely-related species in many taxa, which can result in the inconsistency between mitochondrial and nuclear relationships among these species [45,46]. The second is that there are approximately 20 extant closely-related species in the genus *Takifugu*. Without surveying all of them, it will be difficult to infer the precise timing and direction of transitions between the two sex-determining loci. Taken together, a phylogenomics approach that compares the genomes of the majority of species belonging to *Takifugu* using next-generation sequencing data, will be required to generate a robust phylogeny of *Takifugu* species, facilitating the inference of the ancestral and derived states of the position of sex-determining locus among them.

### Fixation of the presumably hypomorphic allele

Our survey of a wild *T*. *niphobles* population suggests that the *Amhr2* locus is fixed for the C allele at the SNP7271 position (Figs 2 and 3). A previous study implied that fugu Amhr2^H384^ protein encoded by the C allele may mediate less signaling compared to fugu Amhr2^D384^ protein encoded by the G allele due to the single nucleotide difference that converts Asp384 in the kinase domain into His384 [26]. However, it is not likely that the C allele of *Amhr2* gene in fugu or *T*. *niphobles* encodes a complete loss-of-function protein, as no signs of pseudogenization were detected: there were no frame shifts or indels in their deduced coding regions (S5 Fig). In addition, the C allele in fugu shows a tissue-specific expression pattern in the differentiating ovary [26] suggesting that the Amhr2^H384^ protein plays a role in the gonads other than in sex determination *per se*. Consistent with this speculation, Amh/Amhr2 signaling has been shown to regulate the proliferation of germ cells in developing gonads in medaka [47]. Furthermore, the involvement of Amh/Amhr2 signaling in the differentiating teleost testes has also been suggested [48,49]. In the linage leading to *T*. *niphobles* or fugu, the two sex-determining loci may have coexisted. After that, fixation of an allele at the one locus in a population would set the stage for segregation of alleles at the second locus, resulting in a 1:1 sex ratio. This speculation is consistent with the theoretical works suggesting that multilocus systems for sex determination are usually not persistent [50,51].

### Reduction of recombination around the sex-determining locus

Suppression of recombination around a sex-determination locus is a typical feature of evolving sex-chromosomes, and has been hypothesized to occur by a selective pressure to reduce recombination between the sex-determining locus and linked genes with sex-specific effects [52,53]. Our data also provide evidence that the sex-determining locus in *T*. *niphobles* is located in a region where paternal recombination is strongly reduced (Fig 5, S3 Fig and S4 Fig). Although differences in DNA sequence between sex chromosomes (e.g. inversions and the accumulation of Y-specific DNA sequence) have often been assumed to initiate and maintain recombination arrest, such structural differences are not necessarily required to generate sex differences in recombination patterns[54]. For example, genome-wide variation in recombination rate between phenotypic sexes (heterochiasmy) is reported in many species[55]. Indeed, our experiment using the sex-reversed XY female clearly showed an elevation of recombination ratio around the sex-determining locus between X and Y chromosomes in a female when compared to the ratio in a male (S3 Fig). Thus, the reduction of recombination around the sex-determining locus in *T*. *niphobles* males depends on phenotypic sex rather than differences in DNA sequence between a heterogametic sex-chromosome pair (X and Y). Similar dependence on phenotypic sex has been reported for recombination patterns around the sex-determining locus in medaka [56,57]. Because such a region with phenotypic-sex-dependent reduction of recombination can ensure the linkage between the sex-determining locus and its neighborhood genes with sex-specific effects, if any, it should be a good place to house a new sex-determining gene in the process of sex-chromosome turnover.

### Young and small sex-determining region

After recession of recombination around the sex-determining locus, the theory of sex-chromosome evolution predicts that differences in DNA sequence between a heterogametic sex-chromosome pair will accumulate, the non-recombining region will expand, and the degeneration of the genes in the minor sex-chromosome such as Y and W will begin [58]. In the case of *T*. *niphobles*, no dominant Y-specific or X-specific genetic marker was identified in this study. In addition, the crossing experiment demonstrated the viability of YY individuals. The ratio of the three genotypes of viable juveniles (XX:XY:YY = 26:81:33) did not differ significantly from the 1:2:1 ratio expected for transmission from the XY female and XY male parent (*P* = 0.125109, chi-squared test). Therefore, it is reasonable to consider that the degeneration of Y chromosome has not reached the level of severe haploinsufficiency [59,60]. Furthermore, fine mapping narrowed down the sex-determining locus to the interval corresponding to approximately 300-kb of the sequence in the fugu genome (Table 2). Collectively, these data suggest that X and Y chromosomes in *T*. *niphobles* are at a very early phase of sex-chromosome evolution with little sequence diversity, as observed in some non-mammalian vertebrates such as fugu, medaka, and African clawed frog [9,11,26]. Further studies at the genomic sequence level will be needed to determine the extent of sex-chromosome-specific differences in DNA sequence in this species.

### Candidate genes and future genome assemblies

Our search for candidate causative genes in the sex-determining QTL yielded 12 predicted protein coding-genes (Table 2). Although their roles in the sex-determination have not reported, none of them should be excluded as candidates at this time, since a variety of genes can trigger the sex-determination pathway in non-mammalian vertebrates [7,28,61]. However, it is important to note that the segment corresponding to the QTL interval lies in the fugu genome where the reference sequence is highly fragmented and incomplete. In addition, it is possible that the acquisition of X- or Y-specific sequences may be accompanied by the birth of the sex-determining locus in *T*. *niphobles* as reported in other species [9,11,25]. Therefore, obtaining the reference sequences for X and Y chromosomes in *T*. *niphobles* is desirable to identify the gene(s) responsible for sex determination in this species.

Recent advances in next-generation sequencing techniques have made the construction of a high-quality genome assembly faster and less expensive than before. In particular, long-read technology has enabled the production of more contiguous assemblies than from short-reads, and hence has facilitated many studies including the genomics of sex chromosomes [62,63]. Besides aiding the identification of the master sex-determiner(s) in *T*. *niphobles*, contiguous genome sequences should make it possible to characterize X- or Y-specific sequences and to search for genes with sex-specific effects in the proximity of the sex-determiners. Those genes could play a role in the establishment of the sex-determining loci on LG19 in *T*. *niphobles*.

Further characterization of *T*. *niphobles* sex chromosomes should provide opportunities to gain insight in the process sex-chromosome turnover and a role of reduced recombination in it. Moreover, a comparison of *T*. *niphobles* and other closely-related species with the known candidate gene for sex determination, *Amhr2*, provides an opportunity to study the divergent evolution of sex-determination pathways in closely-related species.

## Supporting information

S1 Fig Association test for phenotypic sex and marker genotypes, and quantitative trait loci (QTL) analysis in *T. niphobles* Family 2. (A) Plot of–log_10_ (*P* value) versus chromosome position for association test of *T*. *niphobles*. The chromosomal position of the markers was first inferred from the draft genome sequence of fugu, and later confirmed partially by linkage analysis shown in Fig 4B and S1B Fig. Closed and open circles indicate data from paternally and maternally inherited markers, respectively. Bonferroni correction gave a significance threshold of–log_10_ (*P*) = 2.5 (blue dotted line). The segmented bar next to the–log_10_ (*P*) plot illustrates the sequence map of fugu chromosome 19, in which each segment schematic represents a scaffold in the FUGU5/fr3 assembly [35,36]. (B) Chromosome-wide mapping of sex-determining QTL. Log of odds scores are plotted in the linkage map of *T*. *niphobles* LG19. The blue dotted line indicates chromosome-wide significant (0.1%) levels of log of odds scores, calculated from 10,000 permutations. The red line in the graph indicates 95% Bayesian confidence interval (CI). Genetic markers are ordered and placed based on both the linkage analysis of *T*. *niphobles* (in the graph) and their comparative location in the fugu genome (on the segmented bar). There was no discrepancy in their order at this resolution of linkage analysis. The *Amhr2* locus (red) did not perfectly co-segregate with 95% CI (red line).
(TIF)

S2 Fig Female and male meiotic map of LG (linkage group) 19 in *T. niphobles* Family 2. Allelic bridges are indicated by a line connecting the female (left) and male (right) linkage maps. Genetic distances in centimorgans between adjacent markers are shown. Genetic markers are ordered and placed based on both the linkage analysis of *T*. *niphobles* (in the graph) and their comparative location in the fugu genome (on the segmented bar). There was no discrepancy in their order at this resolution of linkage analysis. Since it was not known if sex-reversed fish were present, two male maps were generated under the two conditions. SD* and SD** denote sex-determining locus.
(TIF)

S3 Fig The search for individuals with recombination near the sex-determining locus. “X” and “Y” indicate female-associated and male-associated alleles, respectively, inherited from the father. Empty blocks indicate non-informative markers. The first row contains marker names. There were six individuals with recombination between the markers f383 and f2004/f714 in the four families (Families 4–7) composed of 502 siblings in total. However, no recombination between the markers f1637 and f714 was observed. Genetic markers are ordered and placed based on their comparative location in the fugu Chr19 (the segmented bar).
(TIF)

S4 Fig Female and male meiotic maps of LG (linkage group) 19 in *T. niphobles* Family 3. Allelic bridges are indicated by a line connecting the female (left) and male (right) linkage maps. Genetic distances in centimorgans between adjacent markers are shown.
(TIF)

S5 Fig A comparison of the *Amhr2* gene between *T. niphobles* and fugu. Coding regions (red letters) were deduced from a comparison between the fugu *Amhr2* gene sequence (fugu_Amhr2) in the fugu Chr19 (FUGU5/fr3, [36]) and its full-length cDNA sequence deposited in DDBJ (accession number AB618627). A comparison of the gene sequence between fugu and *T*. *niphobles* indicated that there is no frame shift or insertions/deletions in the *T*. *niphobles Amhr2* gene (TN_Amhr2). The SNP7271 of fugu *Amhr2* gene and its corresponding site in *T*. *niphobles* are labeled in cyan.
(DOCX)

S1 Table Primer sequences for DNA sequencing. (XLSX)

S2 Table Number of fish with recombination between f383 and f714 in families 4–7. (XLSX)

S3 Table Primer sequences for microsatellite markers and their genomic position. (XLSX)
