# Supplementary figures and images for: Identification of the sex-determining locus in grass puffer (Takifugu niphobles) provides evidence for sex-chromosome turnover in a subset of Takifugu species
Source: PLoS One. 2018 Jan 2;13(1):e0190635. doi: 10.1371/journal.pone.0190635 (PMC5749833; doi:10.1371/journal.pone.0190635)

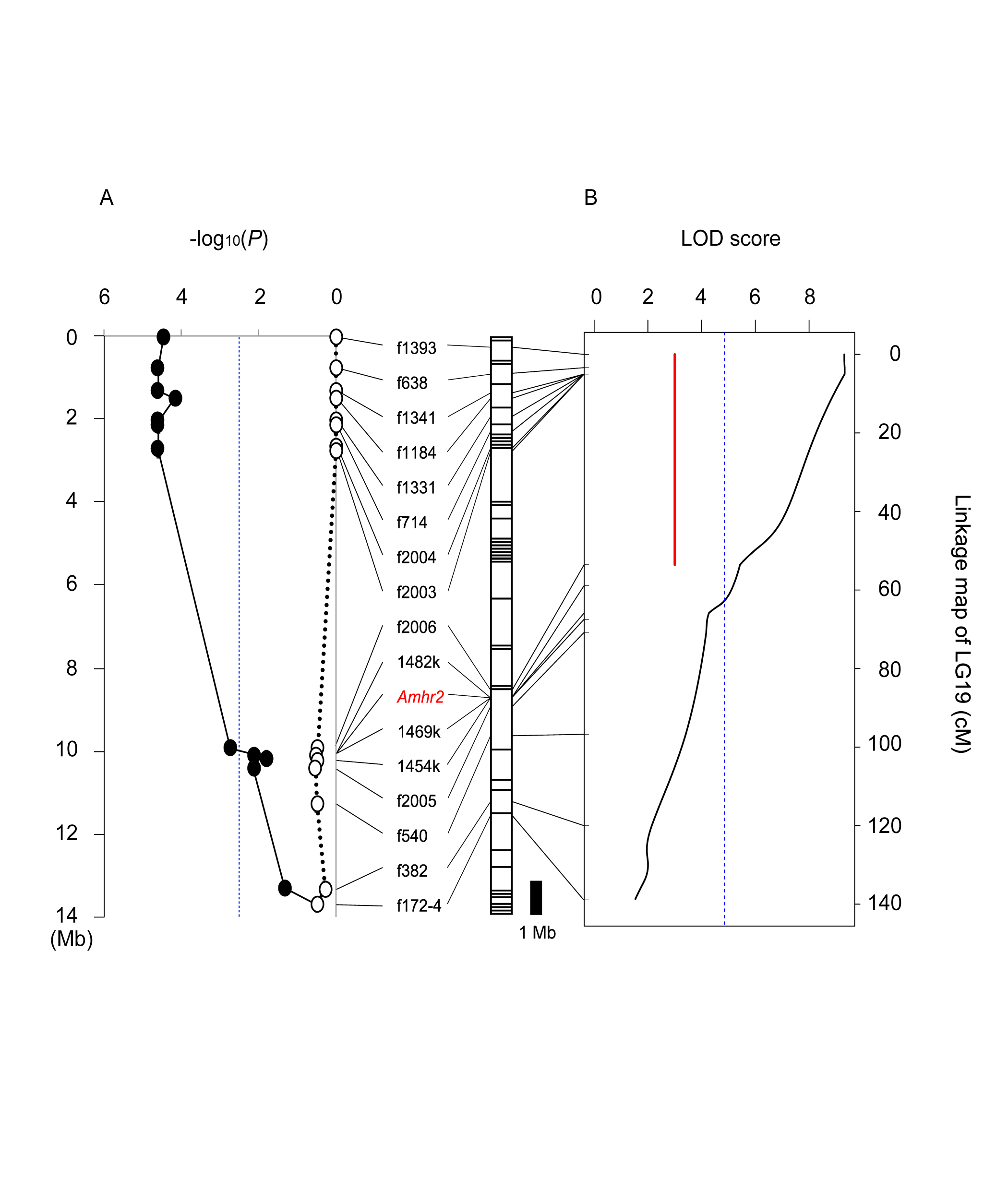

Supplement: S1 Fig — (A) Plot of–log10 (P value) versus chromosome position for association test of T. niphobles. The chromosomal position of the markers was first inferred from the draft genome sequence of fugu, and later confirmed partially by linkage analysis shown in Fig 4B and S1B Fig. Closed and open circles indicate data from paternally and maternally inherited markers, respectively. Bonferroni correction gave a significance threshold of–log10 (P) = 2.5 (blue dotted line). The segmented bar next to the–log10 (P) plot illustrates the sequence map of fugu chromosome 19, in which each segment schematic represents a scaffold in the FUGU5/fr3 assembly [35,36]. (B) Chromosome-wide mapping of sex-determining QTL. Log of odds scores are plotted in the linkage map of T. niphobles LG19. The blue dotted line indicates chromosome-wide significant (0.1%) levels of log of odds scores, calculated from 10,000 permutations. The red line in the graph indicates 95% Bayesian confidence interval (CI). Genetic markers are ordered and placed based on both the linkage analysis of T. niphobles (in the graph) and their comparative location in the fugu genome (on the segmented bar). There was no discrepancy in their order at this resolution of linkage analysis. The Amhr2 locus (red) did not perfectly co-segregate with 95% CI (red line). (TIF) [file pone.0190635.s001.tif]

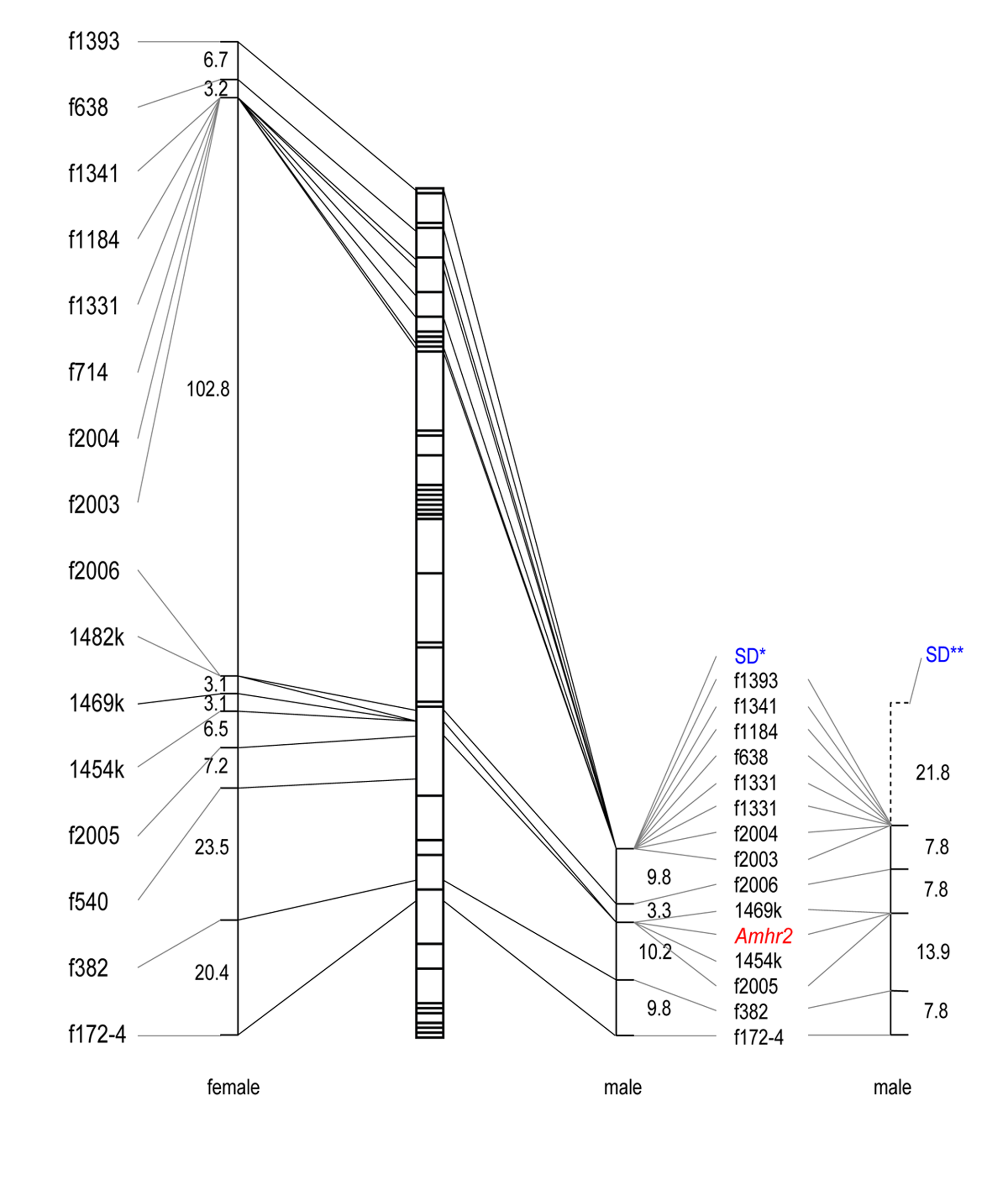

Supplement: S2 Fig — Allelic bridges are indicated by a line connecting the female (left) and male (right) linkage maps. Genetic distances in centimorgans between adjacent markers are shown. Genetic markers are ordered and placed based on both the linkage analysis of T. niphobles (in the graph) and their comparative location in the fugu genome (on the segmented bar). There was no discrepancy in their order at this resolution of linkage analysis. Since it was not known if sex-reversed fish were present, two male maps were generated under the two conditions. SD* and SD** denote sex-determining locus. (TIF) [file pone.0190635.s002.tif]

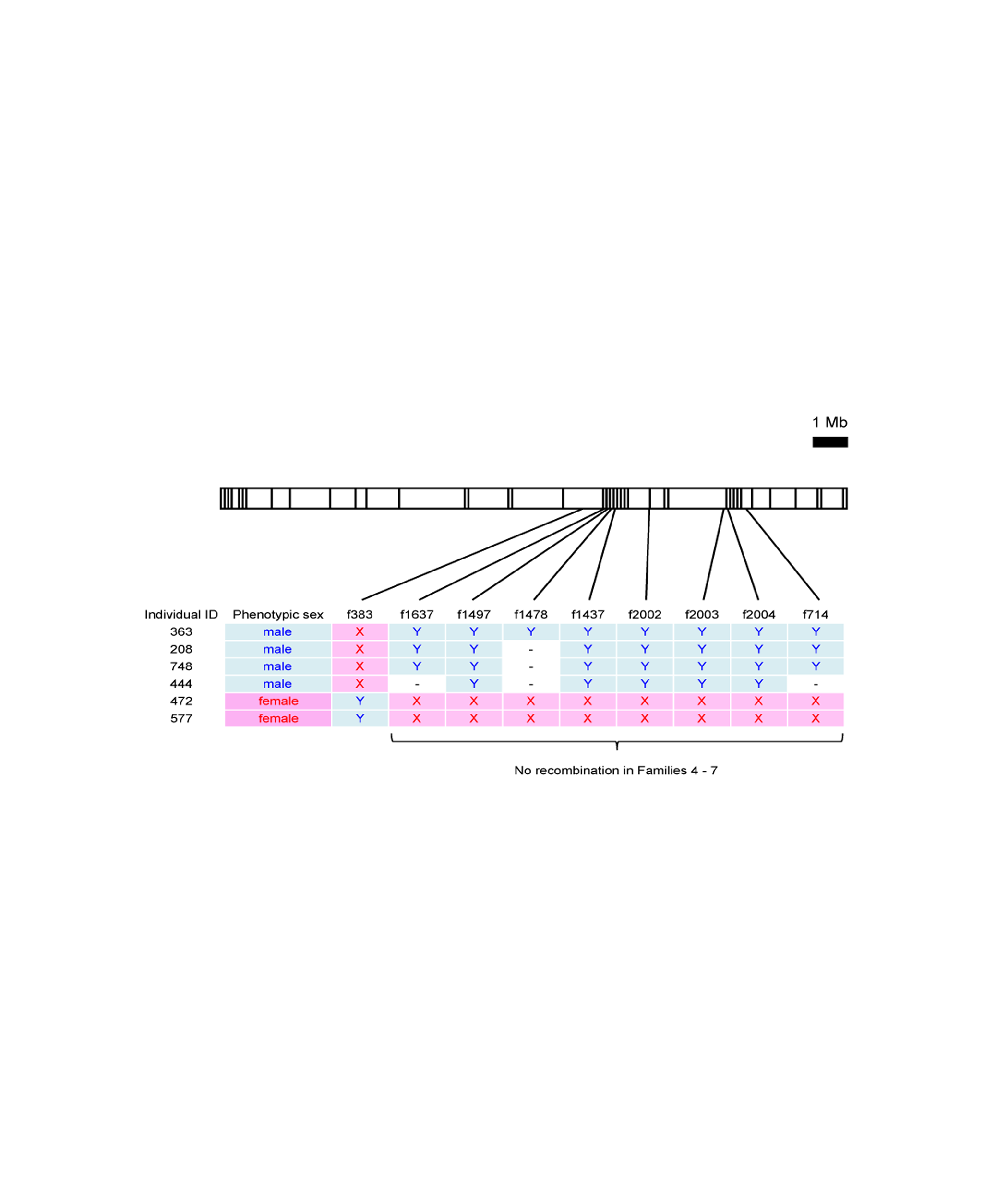

Supplement: S3 Fig — “X” and “Y” indicate female-associated and male-associated alleles, respectively, inherited from the father. Empty blocks indicate non-informative markers. The first row contains marker names. There were six individuals with recombination between the markers f383 and f2004/f714 in the four families (Families 4–7) composed of 502 siblings in total. However, no recombination between the markers f1637 and f714 was observed. Genetic markers are ordered and placed based on their comparative location in the fugu Chr19 (the segmented bar). (TIF) [file pone.0190635.s003.tif]

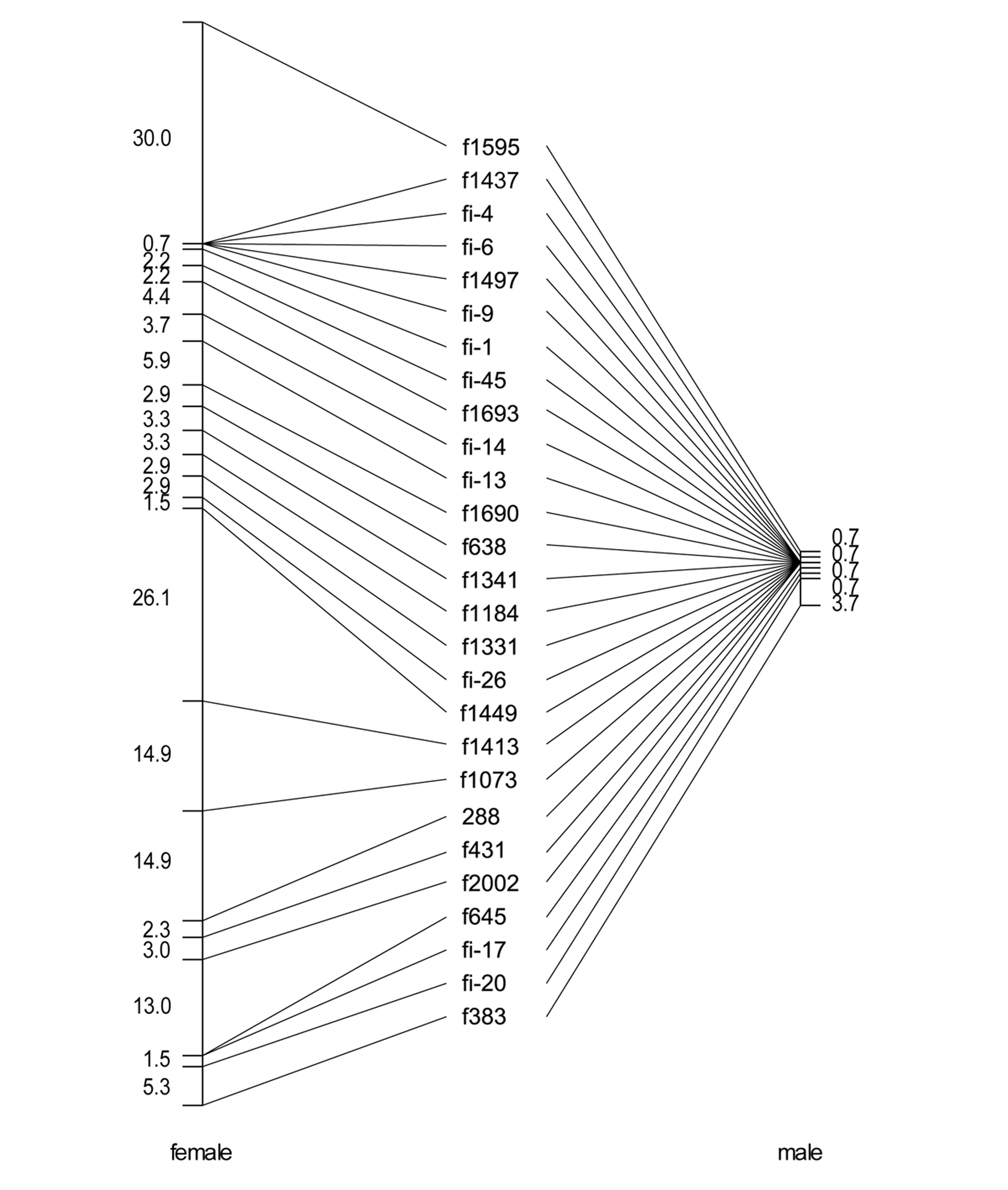

Supplement: S4 Fig — Allelic bridges are indicated by a line connecting the female (left) and male (right) linkage maps. Genetic distances in centimorgans between adjacent markers are shown. (TIF) [file pone.0190635.s004.tif]

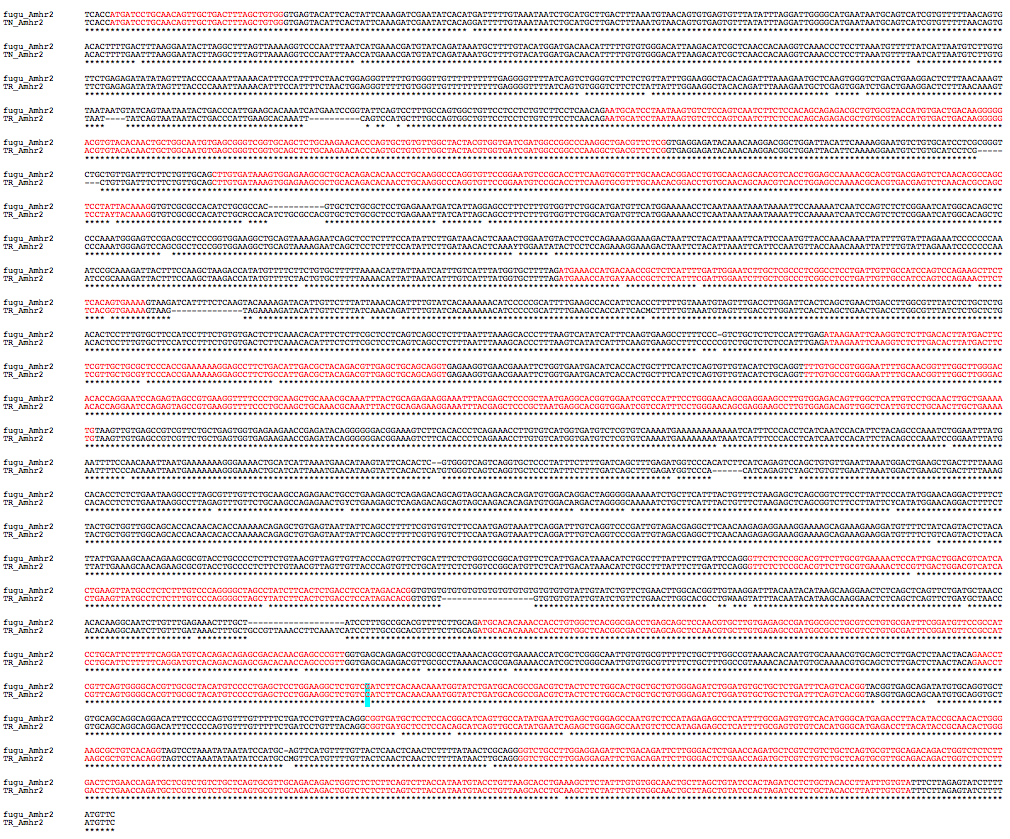

Supplement: S5 Fig — Coding regions (red letters) were deduced from a comparison between the fugu Amhr2 gene sequence (fugu_Amhr2) in the fugu Chr19 (FUGU5/fr3, [36]) and its full-length cDNA sequence deposited in DDBJ (accession number AB618627). A comparison of the gene sequence between fugu and T. niphobles indicated that there is no frame shift or insertions/deletions in the T. niphobles Amhr2 gene (TN_Amhr2). The SNP7271 of fugu Amhr2 gene and its corresponding site in T. niphobles are labeled in cyan. (DOCX) [file pone.0190635.s005.docx]
